# Intra-articular angioleiomyoma of the knee with an atypical finding on gadolinium-enhanced magnetic resonance imaging: a case report

**DOI:** 10.1186/1752-1947-8-238

**Published:** 2014-07-01

**Authors:** Taisuke Fukawa, Yorikazu Akatsu, Takahisa Sasho

**Affiliations:** 1 Department of Orthopaedic Surgery, Graduated School of Medicine, Chiba University, 1-8-1 Inohana, Chuo-ku, Chiba 260-8670, Japan

**Keywords:** Angioleiomyoma, Gadolinium, Intra-articular lesion, Knee, MRI

## Abstract

**Introduction:**

Angioleiomyoma is a benign smooth muscle tumor. It originates in the tunica media of veins. In general, it arises from the dermis, subcutaneous fat and fascia of lower extremities in middle-aged women. The case of intra-articular occurrence is very rare.

**Case presentation:**

This case report describes a 30-year-old Asian man with a 6-month history of recurrent pain localized in the lateral side of his left knee. Magnetic resonance imaging revealed an isolated intra-articular lesion. We performed a surgical excision of this lesion. On histological examination, the diagnosis of angioleiomyoma was confirmed. After the surgery, he recovered completely. Furthermore, no sign of recurrence was observed 1 year after surgery.

**Conclusions:**

In this study, we report a rare case of intra-articular angioleiomyoma of the knee. The mass showed atypical findings on magnetic resonance imaging including gadolinium-enhanced imaging in comparison to previously reported intra-articular lesions. This intra-articular tumor is difficult to diagnose prior to surgery due to paucity of information.

## Introduction

Angioleiomyoma is a rare form of leiomyoma, originating from the tunica media of the vessel walls; it is a benign tumor with a predilection for the lower extremities in middle-aged women. In general, angioleiomyoma is painful and located in the subcutaneous fat and fascia [1,2], but its intra-articular occurrence is extremely rare. To the best of our knowledge, only two such cases have been reported in English, and gadolinium (Gd)-enhanced magnetic resonance imaging (MRI) examination was performed in only one of them [3,4]. The atypical Gd-enhanced MRI image of our case compared with that of the more common angioleiomyoma arising in the subcutaneous fat may provide much preoperative information of this condition.

## Case presentation

**Figure 1:**
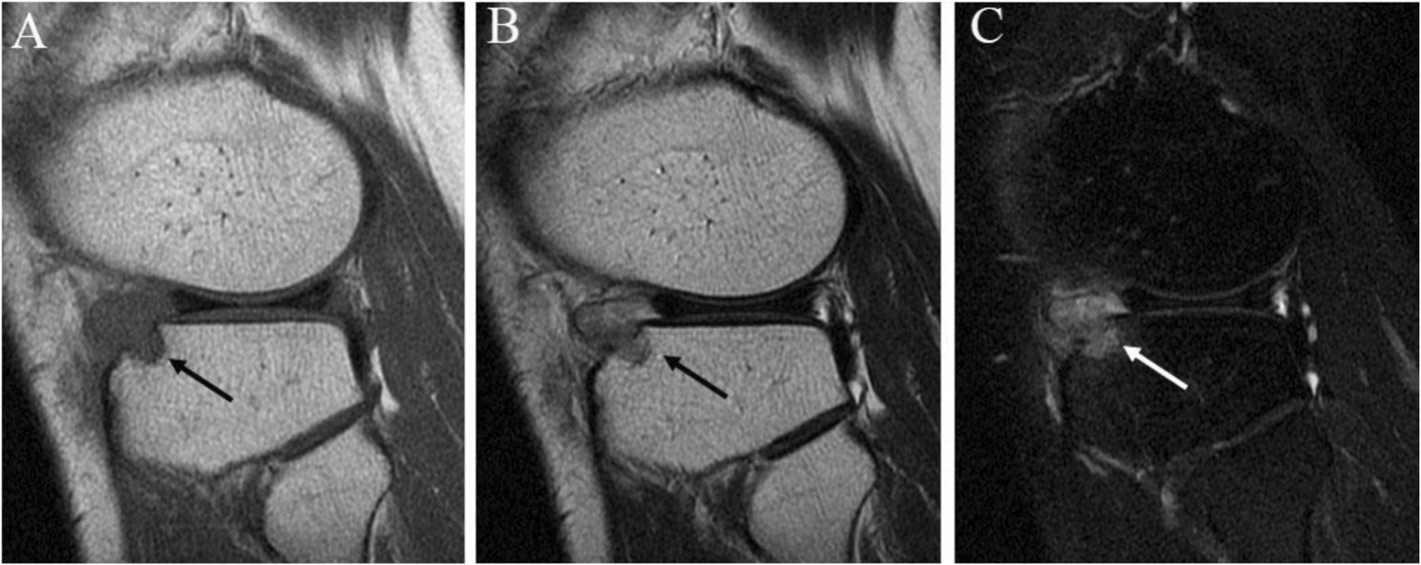
**Magnetic resonance imaging reveals an intra**-**articular mass** (**arrow**) **in the knee joint. (A)** T1-weighted magnetic resonance imaging reveals that the mass is homogeneous and isointense to normal muscle. **(B)** and **(C)** T2 with saturated fat signal sagittal section images show heterogeneous signal intensity with specks. Magnetic resonance imaging: Discovery-MR750 3.0 Tesla (GE Healthcare, Waukesha, WI, USA); T1: repetition time/echo time = 444/10, T2: repetition time/echo time = 3500/90, T2 saturated fat signal: repetition time/echo time = 4400/102, field of view = 160×160mm, slice thickness = 4mm.

**Figure 2:**
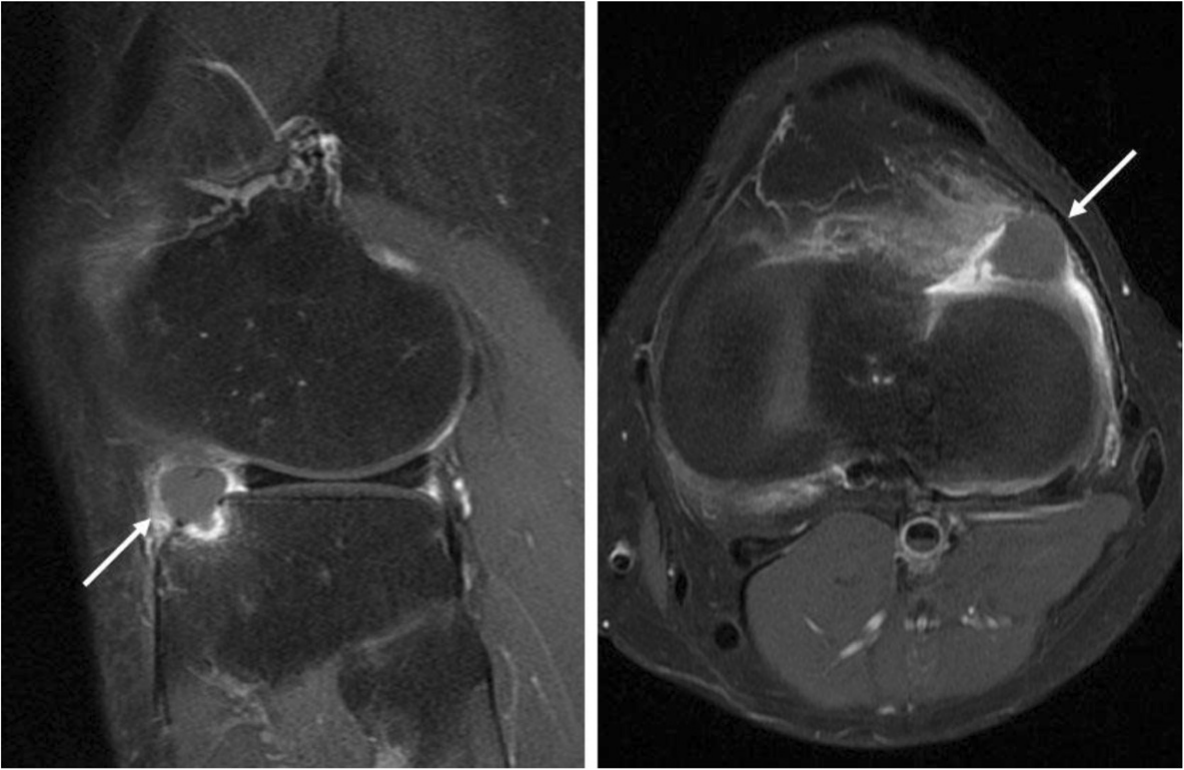
**Gadolinium enhancement with fat saturation magnetic resonance images.** A homogeneously isointense lesion is identified on both sagittal and axial sections of the images (arrows). The mass is enhanced only in the peripheral area. Gadolinium-enhanced magnetic resonance imaging, repetition time/echo time = 558/10, field of view = 160×160mm, slice thickness = 4mm.

**Figure 3:**
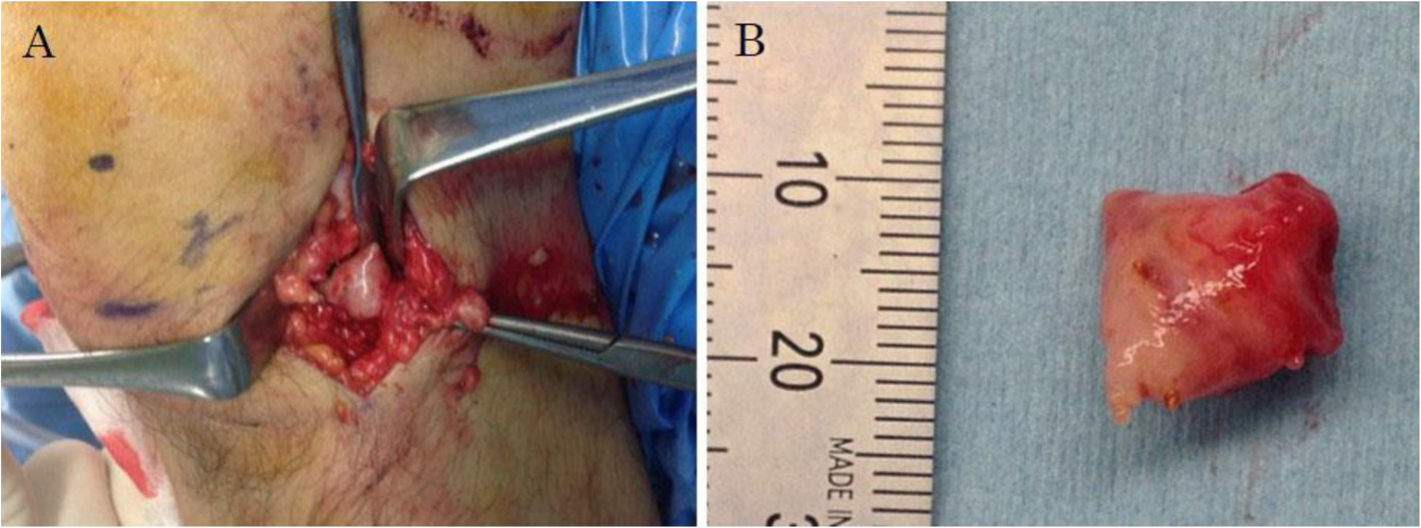
**Clinical appearance of the tumor. (A)** Clinical appearance of the tumor. **(B)** The mass after resection, measuring 15×12×10mm.

**Figure 4:**
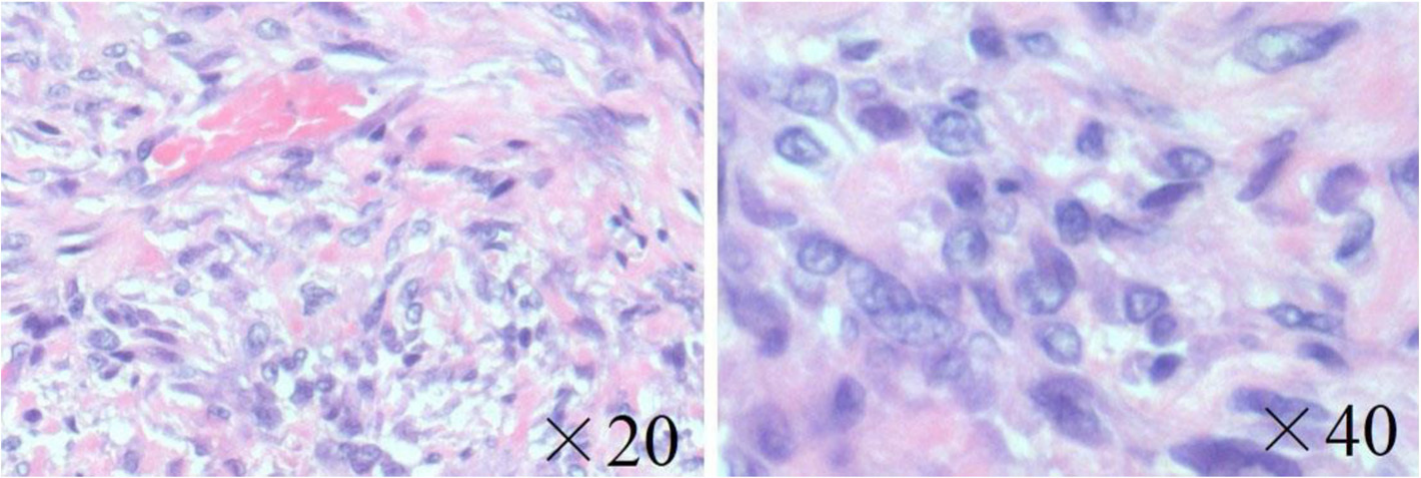
**Microscopic specimen demonstrating an angioleiomyoma.** Microscopic findings reveal that the tumor comprises numerous blood vessels of various sizes and abundant fascicles of smooth muscle bundles surrounding the vessels (hematoxylin and eosin; ×20, ×40).

A 30-year-old Asian man presented with a 6-month history of recurrent pain localized to the lateral side of his left knee. A physical examination revealed slight tenderness and swelling in the lateral side of his knee; however, no other abnormality was noted. Routine radiography showed no remarkable findings, and laboratory data were normal. MRI revealed an intra-articular space-occupying lesion (SOL) in his knee joint, measuring 1.5cm in diameter, which was invading his tibia (Figure 1). The mass appeared homogenous and isointense in contrast to normal muscle on the T1-weighted sequences, and it appeared heterogeneous with specks on T2-weighted sequences. In addition, the mass was unremarkable on observation under T2 with saturated fat signal sequences. Therefore, we performed a Gd-enhanced MRI for differential diagnosis including malignant tumor, and to accurately study the extent of the mass with enhancement being observed only in the peripheral area (Figure 2). No other abnormalities, such as satellite lesions, were detected with the enhancement. Arthroscopic examination was performed to confirm the nature, location and extension of the mass. The tumor was present in the bottom of the anterior horn of lateral meniscus with vascular invasion, but grasp of a total image and total excision using this method was impossible. Therefore, an arthrotomy was performed through a 4-cm skin incision just lateral to the patellar tendon. We performed complete excision of the mass and debridement of the corresponding tibia. A macroscopic examination revealed an oval, smooth-surfaced, soft, and elastic excised mass, which was white and red in color and measured 15×12×10mm (Figure 3). When cut in halves, the tumor was encapsulated. A microscopic examination revealed that the tumor comprised numerous blood vessels of various sizes with abundant fascicles of smooth muscle surrounding the vessels. No atypia was noted in any of the specimens. Histological diagnosis was a benign angioleiomyoma (Figure 4). The patient recovered completely after the surgery. No signs of recurrence were observed up to 1 year after surgery in an MRI examination.

## Discussion

Angioleiomyoma generally presents as a solitary and painful subcutaneous lesion occurring in the extremities, most frequently in the lower limb. The first comprehensive review of this rare tumor was published by Stout in 1937 [2]. To the best of our knowledge, to date, only two cases of intra-articular angioleiomyoma in the knee joint have been reported [3,4]. An angioleiomyoma can clinically resemble a glomus tumor because of episodic focal hyperesthesia, temperature sensitivity, and severe tenderness. However, our case and the previous two cases of intra-articular manifestation did not show these characteristic symptoms. Diagnosis and treatment of these tumors is frequently delayed because of their location and unusual presentation. MRI is a useful tool in diagnosing intra-articular tumors but it is not specific. The typical MRI findings of an angioleiomyoma that originates subcutaneously show an isointense lesion on T1-weighted image and high signal intensity or isointense lesion on T2-weighted image, which was compatible with our case. Previous reports have suggested a homogenous positive enhancement on a T1-weighted image with contrast media. Similarly, in a previous case report of an intra-articular angioleiomyoma with Gd enhancement, homogeneous enhancement of the nodular lesion and heterogeneous signal intensity specks within the lesion were observed. However, in our case the Gd-enhanced MRI finding was atypical, only peripheral enhancement was observed. Based on the conventional MRI findings of our case, differential diagnosis would be localized nodular-type pigmented villonodular synovitis, synovial hemangioma, angiomyoma, giant cell tumor and synovial sarcoma [5-7]. However, even after Gd-enhanced MRI, it was difficult to narrow down diagnosis. The differences in MRI findings may be explained by histological variance; that is, the number of smooth muscle fibers, sinusoidal and dilated vascular spaces, and collagen fibers varied in each case [8]. Alternatively, the intra-articular environment, where the reactive synovial coverage occurs, may have affected the enhancement. These variations were reported in chondromyxoid fibroma, where 69% of them showed peripheral enhancement and 31% homogenous or heterogenous enhancement [9].

Gd-enhanced MRI did not appear to be useful in this specific tumor, but it can still be useful in distinguishing between benign and malignant tumors, evaluating tumor extent, regional metastasis, and identifying satellite lesions. Intra-articular malignant conditions are rare, but cases have been reported such as synovial sarcoma and chondrosarcoma [10,11]. MRI characteristics of synovial sarcoma have been described as follows: triple signal intensity sign (areas of hyper, iso, and hypointensity on T2-weighted sequences), multilobulated or lobulated septate cystic masses with heterogeneous septal, and/or peripheral nodular enhancement [12]. Published studies have identified the following as characteristic MRI features of chondrosarcoma: the tumor reveals low to intermediate intensity lesions on the T1-weighted sequence, possibly with small speckled areas of high signal intensity because of entrapped areas of yellow marrow, and punctate areas of signal void because of matrix mineralization. The signal intensity of non-calcified areas of a tumor was very high on the T2-weighted sequence, with lobules of chondral tissue being separated by hypointense internal septa. Gd-enhanced MRI demonstrates septal and peripheral enhancement of the tumor [13].

To the best of our knowledge, no other cases with SOL in the knee joint, which is compatible with our MRI findings has been reported. Although the precise diagnosis can only be confirmed by histological examination, MRI with Gd enhancement before surgery could be helpful. Excision of the tumor generally results in complete resolution of symptoms, and a recurrence case has not been reported in the literature. The patient in this case recovered completely after the surgery and showed no signs of recurrence at the 1-year follow-up in an MRI examination.

## Conclusions

We describe a rare case of angioleiomyoma that arose in the knee joint. In this case, the symptoms of which the patient complained were different from those of typical subcutaneous angioleiomyoma. Atypical MRI findings in comparison to previously reported cases were observed especially in terms of Gd enhancement. Our patient became completely free of his preoperative symptoms, and no recurrence had occurred at 1 year follow-up. We should be aware of atypical locations of angioleiomyoma as in our case.

## Consent

Written informed consent was obtained from the patient for publication of this case report and accompanying images. A copy of the written consent is available for review by the Editor-in-Chief of this journal.

## Competing interests

The authors declare that they have no competing interests.

## Authors’ contributions

TF and YA performed the surgical procedure and obtained the patient data. TF and TS were major contributors in writing the manuscript. All authors read and approved the final manuscript.
